# Supplementary material for: Are carbon emissions trading and green financial instruments synergistic? -Comprehensive quantitative research based on content analysis
Source: PLoS One. 2024 Mar 7;19(3):e0298601. doi: 10.1371/journal.pone.0298601 (PMC10919601; doi:10.1371/journal.pone.0298601)
Supplement: S1 Data — (ZIP) [file pone.0298601.s001.zip › Data/地级市Z_F工作报告.zip!/╝¬┴╓/╦─╞╜╩╨/└┤╫╘╣1⁄2╓┌║┼í╛┬φ┐╦╩2╛▌═°í┐_2004.docx]

各位代表：

　　四平市第四届人民政府任期五年现已届满。现在，我代表上届人民政府，向大会报告工作，请予审议。

　　一、过去五年工作的回顾

　　过去的五年，市政府紧紧依靠市委的正确领导，自觉接受市人大、市政协的监督支持，团结和带领全市人民，全面贯彻“三个代表”重要思想，励精图治，埋头苦干，奋力拼搏，艰苦创业，圆满地完成了市第四届人民代表大会确定的各项任务。2003年本市生产总值达到230亿元，按可比价格计算，比上年增长14.5%，比1998年的157亿元增长53.9%，年均递增9%。其中一产增加值94亿元，比上年增长10%，比1998年的76亿元增长29.3%，年均递增5.3%；二产增加值65.2亿元，比上年增长23.8%，比1998年的34.6亿元增长94.9%，年均递增14.3%；三产增加值70.8亿元，比上年增长13%，比1998年的47.1亿元增长51.1%，年均递增8.6%。一般预算全口径财政收入16.1亿元，比上年增长10.2%，比1998年的10.66亿元增长51%，年均递增8.6%；市本级一般预算全口径财政收入7.14亿元，比上年增长9.2%，剔除“空转”、“探头粮”等不可比因素，比1998年实际收入的4.14亿元增长72.5%，年均递增11.5%。五年累计完成固定资产投资206.4亿元，比上个五年增长80.1%，年均递增12.5%。争取国债项目93个，到位国债资金27亿元。

　　工业经济整体水平实现新的跨越。坚持工业立市，全面实施项目带动战略，工业经济已经走出低谷，运行质量和效益大幅度提高。2003年全市规模以上工业企业产值达到113.6亿元，比上年增长23.2%，比1998年翻一番，年均递增14.9%；工业企业经济效益综合指数105.7%，比上年提高33个百分点，比1998年提高77.4个百分点；规模以上工业企业利润由1998年亏损5.2亿元到净盈利7477万元，摘掉了连续10年的亏损帽子。项目带动战略取得重大进展。五年累计新上工业项目近1000个，其中投资亿元以上的15个，投资1000万元以上的250个。获国债贴息项目9个，总投资7.6亿元，贴息1.04亿元。总投资1亿元的方向机转向节总成项目已列入国家支持东北老工业基地振兴第一期计划。工业经济结构不断优化。机械制造、农副产品深加工、化工医药等支柱和优势产业产值已占整个工业产值的65%以上；高新技术产业健康发展，实现产值10亿元，是1998年的2.5倍。五年累计完成技改投资44.4亿元，是上一个五年的3倍。全市20家制药企业已有18户进行GMP改造。“双停”企业陆续启动生产，一批资不抵债企业和不符合产业政策的“五小”企业通过关闭、破产退出了市场。

　　农村经济发展迈出实质性步伐。坚持以增加农民收入为核心，以小康建设为目标，农村经济呈现出各业俱兴的喜人形势。农业产业化水平明显提高。黄龙公司、华正公司、红嘴油脂公司、慧泽公司等一批农业产业化龙头企业发展较快，粮食和畜禽加工能力大幅度提高。种植业调整成效显著。大豆发展到192万亩，蔬菜保护地发展到37.7万亩，分别是1998年的8.6倍和4.7倍。花生和专用玉米从无到有，分别发展到43万亩和116万亩。有10个基地和50个产品获得省首批无公害产品基地认证和产品标识认证，获国家级金牌农产品14个，省级名牌农产品136个。粮食生产战胜了以干旱为主的自然灾害，2003年粮食产量达到97亿斤。规模畜牧业快速发展。全市猪、牛、羊、禽饲养量分别达到681万头、209万头、155万只和1.3亿只，比1998年分别增长22.8%、42.8%、33.6%和50.6%，建成各类牧业小区167个。全面推进无规定动物疫病区建设，成为全国首批对世界公布的无规定动物疫病区之一。农业基础设施建设迈上新台阶。五年累计投入水利建设资金4.04亿元，是上个五年的3.4倍，建设水利工程85项。全市完成造林绿化96.1万亩，是上个五年的3.3倍，完成退耕还林还草51.6万亩，实现连续22年无重大森林火灾的目标。二龙湖生态治理全面展开，规划区内和淹没线以下全部实现休耕，共造林5.1万亩，困扰多年的面源污染和生态环境恶化问题基本得到控制。

　　民营经济进入新的发展阶段。高度重视民营经济，全力营造发展环境，不断优化存量、扩张总量，实现了速度效益的同步增长。2003年全市民营经济总产值达到180.8亿元，比上年增长31.7%，是1998年的5.5倍，年均递增40.6%。实现增加值45.9亿元，比上年增长31.7%，是1998年的3.2倍，年均递增26.2%；营业收入超500万元以上的民营企业达到120户，比1998年增加75户；民营经济从业人员达到27.9万人，比1998年增加9.83万人。全市民营经济上缴税金3.8亿元，是1998年的2.3倍，年均递增18.1%，占全市财政收入的23.6%，县域民营经济税收已占当地财政收入的35%以上。公主岭华正公司、一正药业公司等11户企业被评为国家和省诚信企业，民营企业创省以上名牌产品52项。

　　改革开放取得突破性进展。国有企业改革和改组步伐加快。国有企业改制取得显著成效，累计卸掉债务包袱47.8亿元，盘活存量资产20亿元，吸引增量投入10亿元。计划、财税、金融、国土资源、粮食流通体制和城镇住房制度改革取得新的进展。稳定完善土地承包关系，签订了30年土地承包合同。积极稳妥地开展了农村税费改革试点，全市农民合同内负担平均减轻21.1%。市、县、乡三级机构改革顺利完成，各级政府职能进一步转变。招商引资成果显著。共引进各类招商项目2362个，实际到位资金182.33亿元。2003年招商引资实际到位资金45.6亿元，比上年增长35.6%。纳爱斯、巨能、正龙、通钢、福耀玻璃、吉林油田等一批大企业、大集团纷纷来平投资建厂。开发区建设取得了突破性进展。先后引进项目328个，实际到位资金35.29亿元。外贸进出口稳步增长。全市进出口总额3070万美元，比上年增长27.6%。利用外资水平不断提高，累计新批外商投资企业56户，实际利用外资4221万美元。

　　城市面貌发生巨大变化。牢固树立经营城市的理念，采取市场化运作的方式，完成了大量的城市基础设施建设。住宅开发和基本建设取得了突破性进展。五年累计开发402万平方米，是上一个五年的2.1倍。道路建设取得重大进展。累计新建、改造城市主干路10条，次干路、支路88条，新建、扩建桥梁12座，建设街头广场8处，改造土路、断头路167条，修建巷路65万平方米，铺装人行步道70余万平方米，城市核心区域的路网全面改善，四平环城公路已经开工建设。集中力量实施一批精品工程建设。南湖公园经过改造，功能更加完善。筹集资金700余万元，建设较高标准的仁兴商业步行街。全市人民瞩目的英雄广场，通过市场化运作，筹集资金6000多万元，搬迁安置了市政府、市气象局、老干部住宅楼等机关单位和居民，广场面积从7万平方米扩大到10万平方米，成为人们休闲娱乐和健身的理想场所。通过出让站前的1.05万平方米土地开发权，筹集资金5200万元对困扰四平多年的站前广场区域进行了彻底改造，累计投入2.18亿元把站前区域建成新的繁华商业区。南河碧水绿带生态工程基建部分全部竣工，累计投资1.37亿元，建设高标准花岗岩料石护岸工程1万延长米、橡胶坝3处，小游园48处，新增绿地26公顷，铺设污水截流干管5622延长米，使南河成为市区一道亮丽的风景线。大力开展绿化美化。城区累计植树70余万株，栽花1200万株，种草73万平方米，被命名为省级园林城市。建设和完善一批环保工程和城市公共设施。四平污水处理厂建成竣工。城市气化率、热化率分别达到81.9%和80%，空气环境质量达到国家二级标准。城市管理水平全面提高。畅通工程进入全国先进行列，被确定为国家D类二级优秀水平。出台巡警综合执法制度，主要街路实行全天候保洁，开展了清理主次街路占道经营、乱贴乱画、浴池饭店污染扰民、公共场所噪声以及白色污染等专项治理战役，分期分批拆除城市道路和建成区内违法违章建设1.3万余户、24万平方米，按全市统一规划设计的标准，彻底清理整顿市区内各小区的自行车棚，基本消灭了违法违章建设。实行“连带责任追究到尖，整改反馈跟踪到边”的责任追究制度，严厉处罚各种违反城市管理规定的行为。城市清雪工作已经养成习惯，成为全市人民的自觉行动。我市承办了全省人居环境、物业管理等7个方面的现场会，2001年被评为省级卫生城市，2003年被国家命名为国家卫生城市，获得中国人居环境范例奖，在城市建设与管理方面共获得省以上荣誉称号27项。

　　社区建设走在全国前列。按照统一规划、统一设计、统一标准、一次达标的原则，2002年市区建设社区综合用房79处、3.3万平方米，在全国率先实现社区建设全城覆盖的目标，初步形成了城市基层管理、居民服务和民政工作三个平台。确定了适合四平特点的便民服务、救助保障、医疗保健等社区十大功能。民政、文化、卫生、公安、劳动等部门纷纷进入社区，开展了丰富多彩的文化、体育及便民服务等活动。我市先后承办了全国、全省两次城市社区建设现场会并介绍了经验，被评为全国社区建设示范市。国家劳动和社会保障部、省公安厅、省消防局、省劳动和社会保障厅、省计划生育协会分别在我市召开东北华北片企业退休人员社会化管理服务现场会、全省社区警务建设现场会、全省社区消防现场会、全省社区就业现场会、全省社区计划生育工作现场会，总结推广社区建设经验；社区卫生服务、青年活动和消防工作分别在全国会议上介绍经验。2003年，各县（市）建设社区综合用房48处，覆盖率达到100%。城市基层基础功能日趋完善。2003年对市区12个街道办事处、4个基层法庭、2个检察室、2个青年活动中心办公用房全部重新建设；对市区14个公安派出所、各县（市）16个公安分局、各乡镇84个派出所的办公用房全部进行重新建设，总面积达5.17万平方米，在全国率先一次性、全覆盖地解决了派出所用房问题，为公安队伍标准化建设、规范化管理、军事化行动奠定了基础。

　　小城镇建设突飞猛进。到2003年，全市小城镇建设共投入23亿元，新建房屋竣工面积268万平方米，道路、供水、供热等基础设施建设进一步完善。公主岭引卡入岭、火车站调向、东西立交桥工程，双辽城市集中供热、天然气工程，伊通满族博物馆等一批重点工程陆续交付使用，公主岭、梨树污水处理工程先后竣工，双辽污水处理厂、公主岭垃圾处理厂建设进展顺利，城镇环境有较大改善。进入国家级、省级各类试点镇16个，分别在全国小城镇健康发展座谈会和国务院体改办等八部委小城镇建设座谈会上介绍经验，省和国家先后在我市召开小城镇建设现场会。通过小城镇开发建设，拉动了建材、运输、建筑、房地产、餐饮、商贸等相关产业发展。几年来转移农村剩余劳动力1.5万人，有6万多农业人口进入城镇。结合小城镇建设进行了撤乡并镇工作，全市有22个乡镇被合并，减轻了群众负担和财政压力，促进了经济发展。城乡交通基础设施建设实现跨越式发展。累计完成交通基础设施投入21亿元，是上个五年的4.8倍，2003年投入8.2亿元，比上年增长20.8%。累计完成公路建设投入18.4亿元，是上个五年的7.2倍，2003年投入7.65亿元，比上年增长27.4%。公路建设总里程900.4公里，新增公路538.1公里，是上个五年的2.5倍；乡镇通油路率达到100%，比1998年增长33%；行政村通油路率达到35%，比1998年增长12.4%，全市晴雨通车里程达到3362.48公里。

　　社会治安综合治理工作成效显著。始终把维护政治和社会稳定作为工作的重点，严厉打击法轮功等邪教组织活动。超前化解一批不安定因素，妥善处置了一批群体性事件。在为期两年的严打整治专项斗争中，以打黑除恶为重点，深入开展社会治安大排查，集中组织破案攻坚，相继打掉了孙长春、马宏源、曲氏三兄弟等184个流氓恶势力团伙，打处成员620人。打黑除恶、追逃打流、重点治乱、缉枪治爆等四项指标均居全省第一。本着打防结合、预防为主的方针，大力开展打防控系统工程建设，全面实施社区警务战略，积极推广治安有偿承包，初步构建了较为完善的治安防范网络，进一步增强了治安整体防控能力，刑事案件逐年大幅下降，社会治安明显好转，人民群众安全感普遍增强。信访工作取得良好效果。各级政府对信访工作高度重视，用程序化、制度化规范信访工作，稳定了信访形势。通过整顿和规范，市场经济秩序得到明显好转。坚持不懈地抓好安全生产，最大限度减少了安全生产事故的发生。

　　人民生活质量明显改善。城乡居民收入持续增加。2003年城市居民家庭人均可支配收入6707元，比上年增长23.6%，比1998年增长89.8%，年均递增13.7%；农村居民家庭人均纯收入3000元，比上年增长16.5%，比1998年增长22%，年均递增4.1%。城乡居民储蓄存款余额154亿元，比上年增加21.6亿元，比1998年增加49亿元。消费水平明显提高。全社会消费品零售总额77.9亿元，比上年增长10%，比1998年增长32.9%，年均递增5.9%。城镇居民人均住房建筑面积由1998年的8.7平方米增加到2003年的14.3平方米。四平城区的住宅小区全部进行了综合整治，市民生活环境得到较大改善。实行“两个确保”周例会调度制度，使保费由入不敷出到现在可用资金2亿多元，其中市区达1.1亿元。医疗保险参保面达65%，统筹金达到6113万元。就业和再就业工作取得明显成效。全市累计开发就业岗位11.36万个，比上个五年增加2.88万个；安置就业9.67万人，比上个五年增加3.26万人。困难群众的基本生活得到保障。全市城镇低保对象由1998年的2650人增加到2003年的9.6万人，五年累计下拨救灾款4855万元，发动社会捐款捐物2700多万元，解决灾民口粮5.97万吨。

　　科技、教育和各项社会事业全面进步。科技进步与创新步伐加快。五年共取得科技成果145项，其中5项达到国内先进水平。申请国内外专利415项。推进了科研机构与企业的合作，建立企业技术中心96个。教育事业有较大发展。基础教育布局得到优化，教育教学质量明显提高，新建改造校舍21.9万平方米。职业教育和民办教育迅速发展。中高等教育发展有新的突破，四平大学、四平幼儿师范与吉林师大联合办学，建立了吉林师大应用工程学院和吉林师大分院，四平卫校与吉林大学联合办学，建立了吉林大学四平医学院。文化事业进一步繁荣。文艺工作五年来共获国家、省级奖项180余项，社区文化和农村基层文化建设取得较大成绩。广播和电视人口覆盖率分别达到100%和98%。人口与计划生育工作全面发展，人口自然增长率控制在2.6‰。卫生改革成就显著，卫生监督与疾病预防控制体系进一步完善，在全省率先开展药品集中招标采购，医疗服务质量有较大提高。2003年4月中旬，面对突如其来的非典疫情，采取超常规措施，紧紧把住非典输入的“入口关”，严格控制非典传播的“隔离关”，顺利度过了非典疫情的流行期，实现了“三个一”的目标。群众性体育活动广泛开展，竞技体育共获全国冠军30项，我市被评为全国群众体育先进市。旅游投入逐年增多，山门风景区等6个景区被批准为国家2A级旅游景区。妇女儿童、老龄、残疾人、外事、侨务和民族宗教等工作取得新进展，国防教育、国防动员、武装工作、人民防空、军民共建、拥军优属和优抚安置工作不断推进，连续四次获得全国双拥模范城荣誉称号。

　　社会主义精神文明和民主法制建设取得新成果。深入贯彻《公民道德建设实施纲要》，思想道德建设进一步加强。精神文明创建活动深入开展，市中心医院、红嘴集团总公司等8个单位被评为全国精神文明先进单位，我市被评为全国创建文明城市工作先进城市。在全国率先推行了“直选”式的村民自治，政务公开、村务公开、厂务公开取得较大进展。政府自觉接受人大的法律监督、工作监督和政协的民主监督。深化行政审批制度改革，取消各类行政审批项目28项，下放、暂缓执行审批项目10项。成立了政府投资服务中心，开展一站式办公，行政审批效率明显提高。实行大督查责任制度，狠抓工作的落实，实现了政令畅通。各部门克服困难，创造条件，强化措施，完成了大量艰巨的工作任务。认真落实领导干部廉洁自律规定和廉政建设“一岗双责”责任制，坚决纠正部门和行业不正之风，加大查办案件力度，深入推进从源头上预防和治理腐败，各级政府廉政建设取得了较大成效。

　　各位代表，过去的五年是工业经济从艰难中走出低谷、步入良性循环、走向健康发展的五年；过去的五年是各项社会事业齐头并进、竞相发展、全面提升的五年；过去的五年是彻底粉碎黑恶势力、社会治安综合治理取得重大成果、人民群众安全感普遍增强的五年；过去的五年是城市面貌不断改观、城市意识不断增强、人居环境不断改善、人民生活质量不断提高、人口素质发生显著变化的五年；过去的五年是人民政府在改革开放和现代化建设过程中不断提升形象，凝聚力、向心力、吸引力不断增强，四平知名度和影响力不断扩大的五年。

　　回顾五年走过的历程，我们有三点体会：一是解放思想，实事求是。坚持以经济建设为中心，学吃亏，学让利，让利都得利，争利都没利，要想取之，必先予之；你发财我发展，招商引资借助外力发展自己；结合四平实际，理论联系实际，实事求是，因事制宜，因势利导，创造性的落实党的方针政策。二是求真务实，真抓实干。说真话，办实事，不怕苦，不怕累，力戒空谈和形式主义，说了就要算，定了就要办。克难攻坚，勤政为民，心里时刻装着老百姓。咬定青山不放松，不达目的不罢休。三是团结一致，形成合力。找准位置，顾全大局，紧紧依靠市委的领导，维护市委的绝对权威，主动争取人大、政协的监督与支持。政府班子成员齐心协力、九牛爬坡个个出力；政府组成部门围绕中心，服务大局，相互支持，团结协作；全市上下同心同德，齐心协力抓经济。

　　过去的五年，有经验有教训，有曲折的艰辛也有成功的喜悦。我们这一届政府正是受命于四平经济极度困难的历史时期。当时的四平，工业经济滑坡已经到了极限；停产、放假、下岗、待业频频出现；机关的工资不能按月足额兑现；四平人已经丧失了信心，人心涣散，四平人自己贬低自己、自己埋汰自己、自己污辱自己已成习惯，我们这届政府就是处在这样的危机时刻和紧要关头。可见我们这届政府肩负的历史使命、承担的历史责任、承受的工作压力和四平人民对政府的要求与寄托的希望也是可想而知的。五年已经过去，回顾起来，我们觉得我们还算基本上做到了对党和国家负了责，对黎民百姓负了责，对四平的历史负了责。

　　为了四平，为了四平人民，我们不唯书，不唯上，不图官不图名不图利，只图为四平人民干点实事、好事、真事、大事。我们牢固树立了四平市330万人民的最高利益高于一切的思想观念；牢固树立了把四平经济搞上去的强烈愿望压倒一切的思想观念；牢固树立了为官一任造福一方的历史使命超过一切的思想观念。

　　为了四平，为了四平人民，我们宁肯做出牺牲，宁肯牺牲眼前利益，宁肯牺牲一切甚至宁肯牺牲我们这一届，我们也要做四平经济发展历史阶段的奠基人、铺路人和栽树人，为下几届奠基，为未来铺路，为后人栽树，这些是我们这一届一切工作的出发点和落脚点，这是我们过去五年实践的基本指导思想，也就是我们这一届政府的一点体会。

　　五年来所取得的成绩，是市委正确领导的结果，是市人大、市政协监督支持的结果，是全市各级政府和全市330万人民同心同德、团结奋斗、艰苦努力的结果。在此，我代表市人民政府，向全市工人、农民、知识分子、各阶层群众和各级干部，向人大代表、政协委员、各民主党派、人民团体及各界爱国人士，向中省直各单位和驻平部队、武警官兵及公安司法干警，向所有关心和支持我市建设的海内外朋友，表示衷心的感谢和崇高的敬意!

　　各位代表，纵观四平历史，我们前五年确实变化很大。但横看全省其它城市，我们相差甚远，差距很大。这些问题主要是：体制性、机制性矛盾突出，市场化程度低，发展活力不足；经济总量小、结构不优，一产大、比较效益低，二产小、代表先进生产力的企业和装备少，三产水平低、新兴服务业少；经济发展总体上投入不足，吸纳、聚集资本的能力不强；改革开放差距较大，经济发展的外向度过低，对外经济合作和贸易领域不宽，实际利用外资少；人才问题突出，专业技术人才流失严重，接续人才匮乏；农民收入增长缓慢，波动性较大；城镇居民收入水平不高，少数群众和部分行业的一些职工生活还较困难；就业压力仍然很大，社会保障体系尚不完善；各种社会矛盾较多，还存在着一些不稳定因素；政府自身建设有待加强，解放思想，更新观念的任务仍然繁重，依法行政还存在许多问题，形式主义、官僚主义和弄虚作假、铺张浪费现象还时有发生。这些问题，我们要继续采取措施认真加以解决。

　　二、对下届政府工作的建议

　　从现在起到2008年，是我市在老工业基地调整改造中加快发展的关键时期。政府工作总的要求是：以“三个代表”重要思想为指导，全面贯彻党的十六大和十六届三中全会精神，围绕全面建成小康社会的目标，紧紧抓住振兴东北老工业基地和扶持粮食主产区的重大机遇，坚持科学的发展观，充分发挥比较优势和后发优势，坚持工业立市、全面实施项目带动战略，做大做强主导产业和优势产业，以提高经济效益为中心，以结构调整为主线，以改革开放和科技进步为动力，以提高人民生活水平为目的，全面实施四平二次创业，加强社会主义政治文明和精神文明建设，努力实现国民经济跨越式发展和社会全面进步，把四平建设成以主导产业和优势产业为支撑的多产业综合性新型工业城市。

　　主要预期目标是：到2008年，本市生产总值达到430亿元，年均递增13%以上；其中一产增加值年均递增5%左右，二产增加值年均递增20%左右，三产增加值年均递增16%左右。财政收入达到28亿元，年均递增12%。全社会固定资产投资达到90亿元以上。社会消费品零售总额达到137亿元，年均递增12%。城镇居民人均可支配收入和农民人均纯收入分别达到11400元和4120元。人口自然增长率控制在5‰以内，城镇登记失业率控制在5%以内。

　　经过努力奋斗，全面完成“十五”计划，人均本市生产总值在全省位次前移。工业立市、项目带动战略取得重大进展，工业化水平有较大提高，城乡、工农之间差距扩大趋势得到有效遏制；就业状况明显好转，社会保障进一步加强，城乡居民收入较快增长，人民生活质量明显提高；社会主义市场经济体制比较完善，对外开放水平逐步提高；人民民主进一步发展，社会法制比较健全，思想道德建设得到加强，文化事业不断繁荣；生态环境得到实质性改善，可持续发展能力明显增强。总的目标是，将我市初步建成经济繁荣、科技进步、环境优美、社会文明、生活富足的地区，使全市人民过上较殷实的小康生活。?

　　2004年主要预期目标是：本市生产总值增长15%左右，全口径一般预算财政收入增长9.5%；城镇居民人均可支配收入增长10%，农民人均纯收入增长7%；城镇登记失业率控制在5%以内。建议重点做好以下工作：?

　　（一）积极推进工业立市，全面实施项目带动战略，提高产业竞争力。

　　加强项目建设，大力培植新的经济增长点。打破区域界限，围绕国家产业发展重点和周边大城市产业发展方向，扩大协作领域，争取一批国债项目、国内外大集团对外扩张项目和民间投资项目，重点抓好立市立县的大项目、工业项目、高科技项目、农业产业化项目和城市基础设施建设项目。强化市县两级老工业企业调整改造的工作力度，完成技改投入15亿元。加快发展主导和优势产业，培育壮大一批大企业和企业集团。立足我市工业基础优势、交通区位优势等有利条件，壮大机械加工制造配套和绿色农产品加工制造两大主导产业，做强化工、能源、冶金、建材四大优势产业，培育高新技术和现代物流两个新兴产业，改造提升医药、纺织、轻工、电子等传统产业，创造具有四平特色和竞争力的产业新优势。进一步深化改革，增强企业发展的动力和活力。按照建立现代企业制度的要求，引导企业加快经营机制转变，突出抓好困难企业的改制，妥善解决人员分流、债务处理等问题，力争在两年内使国有企业全部实施改制。适时建立统一的国有资产管理机构，依法履行出资人职责，维护所有者权益。

　　（二）加快农业结构战略性调整，建设现代农业，全面实施县域经济突破。

　　抓住国家实施优质粮食工程和专用粮食基地的机遇，建设专用玉米、高油大豆、绿色稻米、优质花生等优势产业带，提高专业化、规模化生产水平。继续狠抓牧业小区建设，新建牧业小区85个，巩固无规定动物疫病区建设成果，打好防治高致病禽流感阻击战，确保动物防检疫安全。加快产业化龙头企业建设，结合国家扶持粮食主产区发展农产品加工业，重点抓好粮豆、畜禽、饮品、中草药、果菜等五大系列44个项目，增强县域经济实力。严格农产品质量管理。落实农产品标准化生产基地372万亩，完成申报认证绿色食品6个，建立无公害产品基地5个，新增无公害产品30个。加强农村基础设施建设。抓好中小型水利工程建设，启动7座小(I)型水库除险加固，新打抗旱井6500眼，探索农业机械化新路子，加快农村公路网建设步伐，改善农业生产条件。大力发展棚膜经济、林业经济，新开发温室大棚6000亩，完成造林绿化20万亩。严格土地管理，切实保护耕地。深化农村各项改革。继续推进农村税费改革，切实减轻农民负担，抓好供销社改革、农村信用社改革、粮食流通体制改革和粮食直接补贴改革。稳定土地承包制度，发展适度规模经营。

　　（三）加大开放力度，拓宽经济发展空间，加速提升开放型经济发展水平。

　　继续抓好招商引资。引进立市立县的工业项目、农业产业化项目、基础设施项目和民营项目，不放松有利于扩大就业和盘活存量的中小项目。在全民招商的基础上，注意提高招商引资的层次和水平。加强招商引资业务培训，提高招商队伍整体素质。大力抓好开发区建设。不断提高其规划、建设水平，完善运行机制和管理体制，落实开发区政策，使开发区真正成为招商引资的载体、对外开放的窗口和经济发展的新增长点。努力抓好对外经贸合作。精心培育一批出口创汇骨干企业，调整出口商品结构，提高竞争力。积极创造条件，吸引国外知名大企业、大集团落户四平。??

　　（四）激发民间活力，放手发展民营经济，着力培育经济增长新亮点。

　　鼓励全民创业。大张旗鼓地营造全民创业的舆论，掀起全民创业的热潮。落实促进民营经济发展的各项政策，把培育个体工商户作为基础性工作切实抓好，在此基础上培育出更多的老板、经理、企业家。积极促进民营企业发展。凡国家法律法规没有明令禁止的领域，都要对民营企业开放。支持民间资本以股份制、合作、独资等多种形式参与基础设施、新型服务业、外经外贸等领域投资。鼓励民营企业抓住国有企业转制的有利时机，通过购买、兼并、联合等形式，参与国有企业改组改造。扶持民营企业围绕我市资源优势和主导优势产业，搞配套加工，实现小配件大配套，小产品大行业，小企业大协作，小资本大聚集，形成集群经济。大力发展现代服务业。重点发展信息、物业、旅游、家政、社区服务等新兴服务业，依托名企、名牌发展连锁经营、物流配送等行业。银行要优先为老工业基地调整改造项目提供资金支持，增加对有市场、有效益、有信誉企业的贷款，规范发展消费信贷。全社会消费品零售总额预期增长10%，居民消费价格总水平提高2个百分点。

　　（五）坚持经营城市理念，提高城市现代化水平，不断改善人居环境。

　　强化规划的先导作用。完善城市总体规划，加强开发区规划的集中统一管理。加快市政基础设施建设。市区重点完成垃圾无害化处理场、火车站站舍改造、北河改造以及污水截流干管二期工程、物流配送中心工程建设。进一步完善路网体系。建设北迎宾街公铁立交桥、南湖大路西段、市府西街、南仁兴商业步行街、铁东三经街商业步行街、英雄大路东段及站前广场工程，完成南环路建设，开工建设北环路。全面提高房地产开发建设水平，争取在建工程达到60万平方米。继续加强绿化美化，扩大绿地面积。重点抓好高标准小区建设，不断提升人居环境质量。强化城市管理工作。加强对土地一级市场的调控，全面实行经营性用地招标、拍卖、挂牌制度。围绕巩固国家卫生城市成果，完善环卫基础设施建设，加强环境综合整治。认真贯彻落实《物业管理条例》。强化房地产市场管理，严格实行资本金制度，绝不允许无资质、缺资金的开发公司进入市场。加强建筑市场管理，搞好建设工程招投标，保证建筑质量。

　　（六）千方百计扩大就业和再就业，健全社会保障体系，切实维护社会稳定。

　　积极做好就业与再就业工作。鼓励发展劳动密集型产业和容纳劳动力较多的服务业，支持国有大中型企业利用非主业资产安置富余人员，发挥个体私营经济在增加就业方面的积极作用。认真落实再就业优惠政策，简化创业审批，减轻税费负担。大力发展城乡劳务经济，拓宽就业渠道。引导全社会转变就业观念，提倡和鼓励自谋职业、自主创业，通过灵活多样的方式实现就业。加强实用技术培训，加快劳动力市场信息化建设步伐。城镇新开发就业岗位5.1万个，新增就业人口4.28万人。不断增强社会保障功能。继续强化社会保险金征徼，多渠道筹集资金，依法扩大社会保险覆盖面，将城镇从业人员纳入基本养老保险。妥善解决好国有及国有控股企业下岗、离岗人员劳动关系及社保关系，加快退休人员社会化管理步伐。进一步扩大医疗保险覆盖面。加强社会救助和帮困工作，实现城镇贫困居民最低生活保障，发展农村社会福利事业。努力维护社会稳定。切实加强信访工作，立足于解决实际问题和把矛盾化解在基层。加大整顿和规范市场经济秩序工作力度。完善安全生产管理监督机制，保障人民群众生命财产安全。进一步加强社会治安综合治理。依法严厉打击各种犯罪活动，有效防范和惩治邪教组织的破坏活动，坚决扫除社会丑恶现象，保持良好的社会秩序。

　　（七）大力发展社会主义先进文化，积极发展社会各项事业，为经济发展提供有力支撑。

　　优先发展教育事业。加强教师队伍建设，推进素质教育，提高教育教学质量。加快普通高中教育发展，整合职业教育资源，发展和规范民办教育、社区教育和职工培训，逐步建立终身教育体系。协调发展文化卫生体育等各项社会事业。积极发展文化事业，弘扬主旋律，提倡多元化，丰富和繁荣人民群众的文化生活。开工建设四平战役纪念馆工程，积极筹建广播电视大楼。继续深化医药卫生管理体制改革，加快建立突发公共卫生事件应急机制、疾病预防控制体系，卫生执法监督体系和医疗救治体系。抓好市传染病医院移址及市急救中心项目建设。搞好新型合作医疗试点、大病统筹和医疗救治工作，改善农村卫生服务条件。稳定低生育水平，加强社区计生工作和流动人口管理，人口自然增长率控制在6‰以内。积极开展全民健身活动，推进群众体育和竞技体育协调发展。加强精神文明建设和民主法制建设。广泛开展群众性精神文明创建活动，树立良好的社会风尚，不断提高市民文明素质和城乡文明程度。加强基层政权建设，搞好村委会第六次换届选举，发挥社区作用，完善社区功能。继续抓好普法教育，深化国防教育，提高国防动员快速转换能力，深入开展“双拥”活动。加强诚信道德建设，加快建立面向个人和企业、覆盖经济社会各个方面的信用体系。

　　（八）加强环境建设，优化经济发展环境，构建文明优质的投资环境。

　　环境是体现一个地方市场经济水平和竞争力的重要标志，是决定四平振兴成败的关键因素。要按照市委“环境建设年”要求，集中整治软环境建设中存在的问题。继续认真解决国家权力部门化、部门权力个人化、个人权力利益化的问题，转变行政观念、行政职能、行政方式、行政行为。集中解决“五个”倾向性的问题，即思想观念陈旧、行政效能低下、政策落实不力、执法水平不高、片面追求部门和个人利益的问题。建立健全环境建设长效机制，实行优化环境的目标责任制和破坏环境的责任追究制，努力创造便捷高效的政务环境、公正规范的法制环境、公平诚信的市场环境、安全稳定的社会环境。

　　（九）加强机关建设，提高行政效率，建设廉洁高效政府。

　　坚持解放思想，实施二次创业。广泛开展“解放思想、抓住机遇、二次创业”大讨论，引导干部群众深刻理解老工业基地调整改造的基本思路和政策原则，坚决破除计划经济的思想束缚，牢固树立主要依靠改革开放实现振兴的思想；坚决破除单纯使用行政手段的思维方式，牢固树立主要依靠市场机制实施振兴的思想；坚决破除消极等靠的思想，牢固树立主要依靠自力更生实施振兴的思想。把干部群众的思想和行动引导到四平二次创业、实现全面振兴的伟大实践中来，走出一条符合市情的振兴之路。坚持服务宗旨，转变政府职能。深化行政管理体制改革，结合事业单位管理体制改革，逐步把政府的专业性、技术性、事务性工作转给事业单位、行业协会和中介组织。抓好行政审批制度改革，大力推行“一站式”办公、“一条龙”服务，为基层和群众办事提供方便。坚持执政为民，改进工作作风。坚决反对和克服形式主义、官僚主义。强化政务督查，确保政令畅通。积极倡导艰苦奋斗、勤俭节约的优良作风，反对铺张浪费、大手大脚的不良风气。坚持从严治政，树立良好形象。依法规范政府行为，推行行政执法责任制，提高依法行政水平。认真落实廉政建设责任制、领导干部离任经济责任审计和述职述廉制度。加强对政府工作人员，特别是各级领导干部的反腐倡廉教育，旗帜鲜明地反对腐败，纠正部门和行业不正之风。

　　各位代表，实现未来五年和今年的各项目标，任务艰巨，使命重大，让我们在市委的正确领导下，高举邓小平理论伟大旗帜，全面贯彻“三个代表”重要思想，与时俱进，开拓进取，团结一致，扎实工作，为加快发展，富民强市，全面建设小康社会而努力奋斗！
